# Blood pressure and proteinuria in older cats and cats with chronic kidney disease

**DOI:** 10.14202/vetworld.2025.527-533

**Published:** 2025-02-27

**Authors:** Maria Natália de Freitas, Maria Sabrina de Freitas, Thaiza Fernanda da Silva, Jéssica Martins Lopes, Juliana Alves Carvelo Nunes Gonçalves, Adriane Jorge Mendonça, Juliano Bortoloni, Pedro Eduardo Brandini Néspoli, Arleana do Bom Parto Ferreira de Almeida, Valéria Régia Franco Sousa

**Affiliations:** 1 Postgraduate Program in Veterinary Science, Faculty of Veterinary Medicine, Federal University of Mato Grosso, Avenue Fernando Correa da Costa, 2367, Boa Esperança, Cuiabá, 78.060-900, Mato Grosso, Brazil; 2 Uniprofessional Residency Program in Veterinary Medicine Faculty of Veterinary Medicine, Federal University of Mato Grosso, Avenue Fernando Correa da Costa, 2367, Boa Esperança, Cuiabá, 78.060-900, Mato Grosso, Brazil; 3 Faculty of Veterinary Medicine, Federal University of Mato Grosso, Avenue Fernando Correa da Costa, 2367, Boa Esperança, Cuiabá, 78.060-900, Mato Grosso, Brazil; 4 Department of Statistics, Federal University of Mato Grosso, Avenue Fernando Correa da Costa, 2367, Boa Esperança, Cuiabá, 78.060-900, Mato Grosso, Brazil

**Keywords:** blood pressure, electrocardiography, feline, kidney disease, proteinuria, older cats, systemic hypertension

## Abstract

**Background and Aim:**

Systemic hypertension and proteinuria are key prognostic indicators in cats with chronic kidney disease (CKD). However, their interrelationship in older cats and those with CKD remains unclear. This study aimed to investigate the association between systolic blood pressure (SBP) and proteinuria in older cats and cats with CKD and evaluate the correlation of these parameters with hematological and biochemical markers.

**Materials and Methods:**

A cross-sectional observational study was conducted on 51 cats divided into three groups: 19 young adult cats (1–6 years, G1), 19 older cats (>10 years, G2), and 13 cats with CKD (creatinine >1.6 mg/dL, G3). Cats underwent clinical evaluations, blood pressure measurements using the oscillometric method, electrocardiography, and hematological, serum, and urine biochemical analyses. Statistical analyses included bootstrapped t-tests and Spearman’s correlation, with significance set at p < 0.05.

**Results:**

SBP values did not significantly differ among groups, but absolute SBP values were higher in older cats (G2), suggesting a potential age-related trend. No significant correlations were found between SBP and proteinuria, creatinine, urea, or urine density in G2 and G3. However, kidney injury biomarkers (urinary protein-to-creatinine ratio, creatinine, and urea) were strongly correlated with weight, body score, and total plasma protein in CKD cats (G3), indicating disease progression. Furthermore, G3 exhibited significant reductions in hematocrit, hemoglobin, and red blood cell counts, which were associated with kidney dysfunction.

**Conclusion:**

This study did not find a direct correlation between SBP and proteinuria in older cats or cats with CKD. However, the higher SBP values in older cats highlight the importance of monitoring hypertension in aging felines. In addition, proteinuria was significantly associated with declining body condition and biochemical markers of kidney dysfunction, reinforcing its value as a prognostic indicator in CKD. Further studies are needed to explore the role of proteinuria and hypertension in advanced CKD stages.

## INTRODUCTION

Systemic arterial hypertension (SH) is defined as a sustained increase in blood pressure (BP) above the reference value. It can be idiopathic, secondary to other diseases, or situational due to stress [1]. Primary or secondary SH is common in older cats [2], particularly in those with chronic kidney disease (CKD) [3].

Unlike dogs and humans, cats with CKD present with interstitial nephritis, tubular atrophy, and fibrosis with secondary glomerulosclerosis [4]. Interstitial fibrosis due to tubular inflammation leads to loss of tubular protein reabsorption and decreased fibrosis. Proteinuria is also considered to be involved in the development of interstitial fibrosis [4, 5]. These processes can result in hypertension and glomerular hypertrophy, leading to proteinuria. Thus, previous studies by Chakrabarti *et al*. [6] and Syme *et al*. [7] have suggested a relationship between creatinine levels, systolic pressure, and proteinuria in cats.

Chronic SH can lead to tissue damage in target organs (eyes, brain, heart, and kidneys), which justify SH monitoring and treatment [1]. Since the kidney is a target organ that can be damaged by SH and CKD is a cause of secondary systemic hypertension, this interaction should always be evaluated, especially in older cats [3]. Therefore, this study investigated the correlation between systolic BP (SBP), which was measured using the oscillometric method, and proteinuria in older cats and cats with CKD. The study also aimed to investigate the correlation of these parameters with other hematological and biochemical findings in older cats and cats with CKD, to assess SH in CKD, and to identify any relationship between SBP, proteinuria, and other renal injury biomarkers, such as creatinine and uremia.

## MATERIALS AND METHODS

### Ethical approval and Informed consent

This study was approved by the Ethics Committee on the Use of Animals of the Federal University of Mato Grosso (23108.000130/2022–63) and received authorization from the guardian of each cat through the signing of an informed consent form.

### Study period and location

From March to October 2023, this observational clinical study was conducted at the University Veterinary Hospital, Cuiabá (15°35’56”S, 56°5’42”W), Mato Grosso, Brazil.

### Animals

The included cats were selected without distinction of breed or sex. A total of 51 cats were divided into three groups: G1, 19 young adult cats aged between 1 and 6 years; G2, 19 older cats older than 10 years [8]; and G3, 13 cats of any age with a history of CKD (creatinine >1.6 mg/dL after hydration), according to the International Renal Interest Society [9].

The exclusion criteria for all groups were aggressive behavior and electrocardiography alterations, as well as comorbidity with CKD or antihypertensive or antiproteinuric therapy for G3.

### Clinical evaluation, BP, and electrocardiogram

The *Cat Friendly* guidelines were followed for the management of each cat [10]. Appointments were scheduled, and on arrival, the cats and their owners were directed to the office to minimize stressful stimuli; 15 min before arrival, a feline facial pheromone was applied to the procedure table and containment towel [10]. The cats were then subjected to clinical examination, including weight measurements, respiratory and heart rates, and temperature measurements.

### SBP, mean arterial pressure (MAP), and diastolic BP (DBP)

DBP was measured using the oscillometric method (petMAP^™^ CardioCommand, Inc., Florida, USA) with a cuff measuring 30%–40% of the circumference of the cat’s thoracic limb [1]. The same observer performed the procedure 6 times; the first value was excluded, and the other five values were averaged.

Electrocardiograph (ECG) was performed using a computerized (ECG Acquisition Module for Computer-Brazilian Electronic Technology, Brazil), and the measurements were performed in bipolar II [11].

### Laboratory analysis

Blood samples were collected by puncturing the jugular, cephalic, or saphenous vein, and urine samples were collected by cystocentesis or spontaneous urination; the cats fasted for 12 h. Blood samples with added ethylenediaminetetraacetic acid were processed by the automated Poche 100 Hematological Analyzer (Sysmex Corporation, Norderstedt, Germany) to obtain hematological values, which were verified by evaluating the blood smear under a light microscope (Leica ICC50 HD, Wetzlar, Germany). Serum and urine biochemical evaluations (urea, creatinine, calcium, phosphorus, protein, and urinary protein: Creatinine ratio [UPC]) were performed using commercial kits in the Wiener® Automated Biochemistry Analyzer (Wiener lab., Rosario, Argentina).

After collection, urine samples were immediately processed for physicochemical analysis using Combur-Test**^®^** (Roche Diagnostic, Mannheim, Germany) reagent strips. Urine density was determined by refractometry (Portable Veterinary Refractometer Model RTV-50ATC, Instrutherm Measuring Instruments Ltd., Brazil), and sedimentoscopy was performed using light microscopy. The hematologic, serum, and urinary biochemical reference values used were those described by Jain [12] and Kaneko *et al*. [13].

### Statistical analysis

Data were recorded and organized in Microsoft Excel, Office 365 (Microsoft Office, Washington, USA) spreadsheets. Statistical analyses were performed using bootstrapped t-tests for independent samples, applying bias-corrected and accelerated confidence intervals to account for small sample sizes and non-normal distributions [14].

Spearman’s correlation was used to evaluate associations between SBP, proteinuria UPC, renal biomarkers (creatinine, urea, and phosphorus), hematological parameters (hematocrit, hemoglobin, and red blood cells), body weight, and body condition score. Correlation strength was classified as weak (r < 0.3), moderate (r = 0.3–0.7), or strong (r > 0.7).

A significance level of p < 0.05 was applied for all statistical tests [15]. Analyses were conducted using R software (version 4.4.2, R Foundation for Statistical Computing, Vienna, Austria) ensuring robust data interpretation.

## RESULTS

In total, 51 cats were evaluated: 19 young adult cats (G1), 19 senior cats (G2), and 13 cats with CKD (G3). The cats comprised 49 mixed-breed (96.08%) and two Persian cats (3.92%), one each from G1 and G3. The mean values for the demographic and clinical variables of the cats and the CKD stage distribution within the groups are presented in Table 1. Among these variables, there was a difference in age and body score. G3 had a lower mean value than G1 (p = 0.004) and G2 (p < 0.001).

**Table 1:** Mean and standard deviation values for the demographic and clinical variables of young adult cats (G1), senior cats (G2), and cats with chronic kidney disease (G3).

Variables	G1 (%)	G2 (%)	G3 (%)
Age (months)	35.84^a^ (21.33)	136.05^b^ (18.23)	84.76^c^ (41.60)
Weight (kg)	4.29 (1.01)	4.52 (1.26)	3.97 (1.21)
Body condition score (1–5)	3.21^a^ (0.41)	3.31^a^ (0.58)	2.53^b^ (0.51)
Respiratory rate (16–20 RR)	22.73 (9.24)	20.84 (5.59)	22.15 (6.45)
T° (37.5°C–39.2°C)	38.13 (0.54)	38.05 (0.52)	38.13 (0.73)
Sex			
Male	68.42 (13/19)	52.63 (10/19)	61.53 (8/13)
Female	31.57 (6/19)	47.36 (09/19)	38.46 (5/13)
CKD stage			
II	-	-	53.84 (7/13)
III			38.46 (5/13)
IV	-	-	7.69 (1/13)

a, b, and c The letters followed by the same lowercase letter do not differ statistically per the *bootstrap* t-test at a 5% significance level. T°=Body temperature (°C), CKD=Chronic kidney disease

The clinical signs of CKD cats were muscle mass loss/weight loss (6/46.15%), dull coat/hair loss (8/61.53%), vomiting (7/53.84%), lethargy (2/15.38%), polyuria (3/23.07%), polydipsia (5/38.46%), halitosis (4/30.76%), dehydration (4/30.76%), hyporexia/anorexia (6/46.15%), and pale mucous membranes (3/23.07%), while cats in the other groups, young and senior cats, did not present clinical alterations, as this was one of the inclusion criteria.

**Figure 1:**
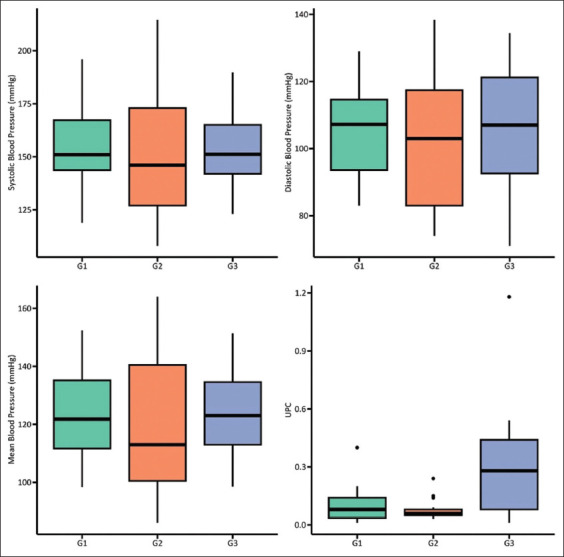
Box plots of systolic, mean, and diastolic blood pressures (BP) and proteinuria (UPC) in young adult cats (G1), older cats (G2), and cats with chronic kidney disease (G3). No significant difference was observed in systolic, mean, and diastolic BP (p > 0.05). Statistical significance was observed for the UPCs between G1 and G3 (p = 0.012) and between G2 and G3 (p = 0.002).

The cats demonstrated sinus rhythm. The mean values of the QRS complex (<40 ms) (electrocardiography deflection that represents ventricular contraction) were above the reference value, with a significant difference between G3 and G2 (Table 2). The mean values of the SBP, DBP, and MAP did not differ significantly between the groups; however, the SBP was above the normotensive range in all groups (Figure 1), and G2 had the highest absolute values.

**Table 2:** Mean and standard deviation values for electrocardiographic findings* and systolic blood pressure (SBP) of young adult cats (G1), senior cats (G2), and cats with chronic kidney disease (G3).

Variables (reference range)	G1	G2	G3	p1	p2	p3
Heart rate (140–220 bpm)	184.64 (29.76)	187.08 (13.90)	184.56 (19.69)	0.797	0.994	0.733
Wave *P* (<35 ms)	32.23 (2.98)	35.55 (5.37)	31.50 (3.25)	0.069	0.604	0.077
Wave *P* (<0.2 mV)	0.08 (0.02)	0.12 (0.14)	0.07 (0.03)	0.244	0.613	0.322
Interval P-R (50–90 ms)	64.62 (6.31)	70.82 (13.01)	70 (7.07)	0.142	0.085	0.874
Complex QRS (<40 ms)	42.62 (5.39)	45^a^ (6.87)	38^b^ (5.07)	0.351	0.067	0.026
Then R (<0.9 mV)	0.32 (0.09)	0.27 (0.13)	0.37 (0.12)	0.255	0.311	0.109
Interval QT (160–220 ms)	169.83 (11.97)	167.27 (21.55)	162.38 (15.21)	0.725	0.236	0.590
SBP (<140 mmHg)	155.66 (20.73)	150.91 (29.43)	155.18 (20.72)	0.600	0.944	0.662

^a, b, and c^Means followed by the same lowercase letter do not differ statistically per the *bootstrap* t-test at a 5% significance level. p1=G1 and G2 comparison, p2=G1 and G3 comparison, p3=G2 and G3 comparison. *Electrocardiography obtained from 14 G1, 12 G2, and 9 G3 cats. SBP=Systolic blood pressure

The mean erythrogram and serum and urinary biochemical values for G3 were statistically significant (Table 3). The mean values for leukocytes and platelets were within the reference values and without statistical significance for all groups. The total plasma proteins showed a statistical difference in G3 (p = 0.02) compared with the other groups; the mean in G3 was slightly above the reference parameter for the species (8.53 ± 1.23). No correlation was observed between SBP and UPC, urine density, creatinine, and urea levels, or age in G2 and G3.

**Table 3:** Mean and standard deviation values of hematological, biochemical, and urinary parameters of young adult cats (G1), senior cats (G2), and cats with chronic kidney disease (G3).

Variables (reference range)	G1	G2	G3	p1	p2	p3
Red blood cells (5.0–10×10^6^/µL )	10.14^a^ (1.17)	9.17^b^ (0.94)	7.47^c^ (1.95)	0.004	<0.001	0.008
Hematocrit (24%–45%)	44.64^a^ (5.28)	39.47^b^ (4.15)	34.23^b^ (9.23)	0.006	0.002	0.082
Hemoglobin (08–15 g/dL)	13.88^a^ (1.52)	12.61^b^ (1.21)	10.93^b^ (3.06)	0.006	0.018	0.082
Urea (42.8–64.2 mg/dL)	46.36^a^ (6.20)	51.78^b^ (9.21)	114.30^c^ (65.31)	0.028	<0.001	<0.001
Creatinine (0.8–1.6 mg/dL)	1.36^a^ (0.17)	1.35^a^ (0.17)	3.11^b^ (1.36)	0.856	<0.001	<0.001
Calcium (6.2–10.2 mg/dL)	10.60 (0.75)	10.78 (1.15)	11.50 (2.66)	0.594	0.170	0.334
Phosphorus (4.5–8.1 mg/dL)	4.44^a^ (1.06)	3.89^a^ (0.64)	5.71^b^ (2.40)	0.058	0.028	0.002
Urine specific gravity (1,035–1,060)	1,056.74^a^ (10.03)	1,055.47^a^ (9.48)	1,024.62^b^ (15.75)	0.700	<0.001	<0.001
UPC (<0.2)	0.098^a^ (0.09)	0.079^a^ (0.05)	0.305^b^ (0.31)	0.438	0.012	0.002

a, b, and c Means followed by the same lowercase letter do not differ statistically per the *bootstrap* t-test at a 5% significance level. p1=G1 and G2 comparison, p2=G1 and G3 comparison, p3=G2 and G3 comparison. UPC=Urine protein-to-creatinine ratio

In G2, there were moderate and significant correlations between age and hematocrit (r = 0.457) and total plasma protein (r = 0.577), UPC and creatinine (r = −0.551) and leukocytes (r = 0.548), calcium and hemoglobin (r = 0.563), and hematocrit (r = 0.631) (Table 4).

**Table 4:** Spearman’s correlation between age, body score, weight, creatinine, urea, calcium, and phosphorus, USG, UPC with erythrogram, TPP, and SBP for cats with chronic kidney disease (G3).

Variables	Red blood cells		Hemoglobin		Hematocrit		TPP		SBP	
				
	r	p	r	p	r	P	r	p	r	p
Age	−0.23	0.443	−0.26	0.390	−0.32	0.278	0.19	0.514	0.07	0.810
Body score	0.49	0.084	0.49	0.084	0.53	0.058	−0.71	0.007	−0.33	0.277
Weight	0.42	0.152	0.41	0.157	0.46	0.110	−0.58	0.036	−0.49	0.089
Creatinine	−0.62	0.023	−0.61	0.028	−0.64	0.018	0.40	0.174	0.47	0.103
Urea	−0.71	0.006	−0.74	0.004	−0.78	0.002	0.52	0.066	0.20	0.500
Calcium	−0.45	0.120	−0.45	0.125	−0.47	0.104	0.20	0.508	0.14	0.641
Phosphorus	−0.67	0.013	−0.67	0.012	−0.72	0.006	0.59	0.032	0.41	0.162
USG	0.75	0.003	0.77	0.002	0.76	0.002	−0.72	0.006	−0.04	0.893
UPC	−0.78	0.002	−0.78	0.002	−0.77	0.002	0.87	<0.001	0.07	0.796

USG=Urine specific gravity, UPC=Urine protein-to-creatinine ratio, TPP=Total plasma proteins, and SBP=Systolic blood pressure

## DISCUSSION

Regarding age, it was expected that there would be no significant difference between G2 and G3 because age is a risk factor for CKD, which is one of the most frequently diagnosed diseases in senior cats and occurs in up to 40% of cats older than 10 years [16, 17].

Cats in group G3 had the lowest body scores and greatest weight loss, with 46.15% of the cats exhibiting a low body score. In this group, these variables showed an inverse correlation with proteinuria, serum phosphorus, and total plasma protein, indicating that cats with proteinuric CKD tend to have lower weights and body scores. Weight loss in cats with CKD is observed in the more advanced stages of the disease, especially when proteinuria becomes evident [18, 19]. Previous studies have reported that proteinuria is a marker of progression in the more severe stages of CKD, reflecting the kidneys’ inability to maintain protein filtration balance [6, 20]. In addition, hyperphosphatemia is commonly observed in cats with CKD and is strongly associated with disease progression [5, 18]. Increased urea concentration has also been correlated with CKD progression [20], as the accumulation of nitrogenous products in the blood reflects the loss of renal function and contributes to the decreased appetite and weight loss seen in the advanced stages of the disease [17, 20].

Despite the mean values of the QRS complex being slightly above the reference in G1 and G2, and significant difference in G3, the complexes did not present an aberrant morphology and were not >60 ms, which indicates a right bundle branch block [21].

The mean values of erythrocytes, hematocrit, and hemoglobin showed a statistically significant difference between G1 and G3, with mean values decreasing as G1 <G2 <G3, although they were still within the normal range in all groups. The reduction of these variables in cats with CKD may be associated with anemia due to the reduction in erythropoietin, a decrease in the lifespan of red blood cells due to uremia, or even functional iron deficiency [4, 22–25]. This corroborates the findings observed in group G3, in which the biomarkers for kidney injury (urine density, UPC, urea, and creatinine) and hematological variables (erythrocytes, hemoglobin, and hematocrit) had strong and significant correlations, except for creatinine, which had a moderate correlation with hematological variables. Decreased hematocrit levels are associated with CKD progression, but they do not predict the development of azotemia in cats with CKD [6].

Cats with CKD may present with chronic systemic inflammation [25, 26], independent of the CKD etiology, which acts on the perpetuation of renal fibrosis and proteinuria [5]. This may explain the higher mean total plasma protein in G3 and the strong correlation between this variable and proteinuria and urinary density. Chakrabarti *et al*. [27] found a positive correlation between proteinuria and interstitial fibrosis scores in cats.

Proteinuria is directly correlated with the magnitude of BP, and hypertension is a risk factor [3, 7]. Glomerular disease (glomerulonephritis) is the most common kidney injury associated with CKD in dogs, whereas diffuse tubulointerstitial nephritis is the most common in cats; thus, proteinuria is less common. Up to 66% of cats with CKD are not proteinuric [7, 17, 18, 28]. Proteinuria occurs in the most advanced stages of kidney disease and is related to the worst prognosis [18, 29], which may explain the results of proteinuria in the borderline range in G3 because only one cat was in stage IV and the majority (53.84%) were in stage II.

Regarding urinary density, G3 was significantly lower than in the other groups. Cats with dehydration and/or azotemic CKD have a urinary density of <1,035 [9, 17, 30]. However, in the early stages of CKD, cats may have hypersthenuric urine [8], even with azotemia [9, 31], because they can still maintain the ability to concentrate urine, which gradually reduces as CKD progresses [32]. Thus, although urinary density is an important marker of CKD, urinary density should not be considered in isolation in the clinical assessment and diagnosis of cats [9, 33].

SBP in all groups was above the normotension values, without differences between the groups; however, this variable was not correlated with proteinuria, even in G3. CKD is one of the leading causes of secondary hypertension [3], and age is considered a risk factor for hypertension, a common finding in senior cats [4, 17]. However, other studies have found no relationship between increased BP and advanced age [1]. Chakrabarti *et al*. [27] observed a correlation between hypertension and proteinuria, although it has not been confirmed whether proteinuria would lead to hypertension or *vice versa*, since proteinuria can be both a cause and consequence of tubular loss, which leads to glomerular hypertension and consequently more proteinuria. Studies have shown that hypertension can occur at any stage of kidney disease and has no relationship with serious creatinine concentration [1], which explains the absence of statistical significance in this study.

Although management according to the *Cat Friendly* guidelines [34] and cat adaptation strategies were employed before BP measurements were obtained, the G1 group SBP values were above the normal range (140 mmHg) [1]. Stress may have been responsible for such values because the hospital environment continued to be a stressor, and the “white coat” effect can increase cat BP by up to 17.1 mmHg with individual changes that can occur up to 80 mmHg, as observed by Belew *et al*. [35].

## CONCLUSION

This study evaluated the association between SBP and proteinuria in senior cats and cats with CKD while assessing their correlation with hematological and biochemical markers. The findings indicate that while SBP values did not significantly differ among groups, senior cats exhibited higher absolute SBP values, suggesting a potential age-related trend. Proteinuria was significantly associated with declining body condition, increased serum phosphorus, and elevated total plasma protein levels in CKD cats. However, no direct correlation was found between SBP and proteinuria, creatinine, or urea levels in either senior or CKD-affected cats.

A key strength of this study is its well-structured cross-sectional observational design, which included both healthy and diseased feline populations for comparison. The comprehensive assessment of multiple physiological parameters, including BP, ECG, hematological, serum, and urinary biomarkers, provided valuable insights into the systemic effects of CKD. In addition, strict adherence to standardized feline-friendly handling protocols minimized stress-related biases in BP measurement.

Despite these strengths, some limitations should be acknowledged. The sample size was relatively small, particularly in the CKD group, which may have limited the detection of weaker associations. The absence of cardiac and renal ultrasound evaluations prevented a more detailed assessment of potential comorbidities such as cardiac disease or structural kidney abnormalities. Furthermore, the hospital setting may have influenced BP readings despite efforts to minimize stress using feline-friendly handling methods.

Future research should focus on longitudinal studies to explore the progression of hypertension and proteinuria over time in aging cats and those with CKD. Incorporating advanced diagnostic tools, such as Doppler BP monitoring, renal imaging, and cardiac assessments, could provide deeper insights into underlying pathophysiological mechanisms. Further studies should also evaluate the impact of antihypertensive and antiproteinuric therapies on clinical outcomes in hypertensive and proteinuric CKD cats.

Although no direct correlation was found between SBP and proteinuria in the studied feline populations, the findings highlight the importance of monitoring hypertension, body condition, and renal biomarkers in senior and CKD-affected cats. These parameters remain crucial in early disease detection and management to improve feline health outcomes.

## AUTHORS’ CONTRIBUTIONS

MNF: Data analysis and drafted the manuscript. MSF and JACNG: Communicated with owners of dogs and collected samples. TFS, JML, and PEBN: Collection of the cat samples. AJM: Laboratory analysis. JB: Statistical analyses. ABPFA: Supervised and revised the study. VRFS: Designed the study and reviewed the manuscript. All authors have read and approved the final manuscript.
